# The Efficacy of Anti-VEGF Therapy for Putative or Visible CNV in Central Serous Chorioretinopathy by Optical Coherence Tomography Angiography

**DOI:** 10.1155/2022/1272524

**Published:** 2022-09-28

**Authors:** Yumeng Zhang, Jingfa Zhang, Xiaodong Sun

**Affiliations:** ^1^Department of Ophthalmology, Shanghai General Hospital (Shanghai First People's Hospital), Shanghai Jiao Tong University, School of Medicine, Shanghai 200080, China; ^2^National Clinical Research Center for Eye Diseases, Shanghai Key Laboratory of Ocular Fundus Diseases, Shanghai Engineering Center for Visual Science and Photomedicine, Shanghai Engineering Center for Precise Diagnosis and Treatment of Eye Diseases, Shanghai 200080, China

## Abstract

**Purpose:**

To investigate the effect and underlying mechanism of intravitreal antivascular endothelial growth factor (VEGF) in patients with putative or visible choroidal neovascularization (CNV) secondary to central serous chorioretinopathy (CSCR) with optical coherence tomography angiography (OCTA).

**Methods:**

In a retrospective cohort study, 16 eyes of 15 treatment-naïve CSCR patients were included and divided into two groups: a putative CNV group with nonhomogenous hyperreflectivity in the slab of choriocapillaris and a visible CNV group with obvious tangled vascular network in the slab of choriocapillaris in OCTA. Patients were recorded with best-corrected visual acuity (BCVA). The parameters, evaluated by OCTA, included central macular thickness (CMT), the height of subretinal fluid (SRF), the number of hyperreflective foci (HRF), and the area of putative or visible CNV.

**Results:**

Compared with the baseline, visual acuity was improved significantly at the last follow-up, and CMT and the height of SRF were decreased significantly (*P* < 0.0001). The number of HRF was also declined in the outer retina and the choriocapillaris layer (*P*=0.0343). Although the visible CNV area in the eyes represented virtually unchanged during anti-VEGF treatment (*P*=0.4015), the area of putative CNV displayed an obvious reduction (*P*=0.0081).

**Conclusion:**

Anti-VEGF is effective in treating CSCR coexisting putative or visible CNV. Early initiation of anti-VEGF therapy benefits CSCR patients with putative CNV detected by OCTA. *Translational relevance* nonhomogenous hyperreflectivity in the choriocapillaris layer in OCTA indicates putative CNV in patients with CSCR, implying early treatment with anti-VEGF.

## 1. Introduction

Central serous chorioretinopathy (CSCR) is characterized by serous subretinal fluid (SRF) accumulation and neurosensory retinal detachment secondary to hyperpermeability from choroidal vessels [1], which is now considered to belong to the pachychoroid spectrum disease [2]. Patients with CSCR are usually between the ages of 20 and 45, accompanied by a sudden decrease of central vision, central scotoma, or micropsia [3]. This disorder is usually self-limited, although in some cases, due to persistent SRF accumulation or multiple recurrences, patients are left with permanent visual impairment because of the damage of the retinal pigment epithelium (RPE) and photoreceptors [4]. Therefore, complete elimination of SRF is of great importance for the CSCR treatment [5].

Choroidal neovascularization (CNV) is a relatively rare complication of CSCR with an estimated incidence of 2% to 15.6% [6], but it is one of the most severe complications of CSCR, which may cause irreversible vision loss [7]. Although CSCR is usually treated conservatively, the development of CNV may require antivascular endothelial growth factor (anti-VEGF) therapy pertinently [8]. Anti-VEGF is currently the empirical treatment for patients with CNV secondary to CSCR, but it is not entirely clear whether the source of SRF is from neovascular leakage [9, 10]. Recently, optical coherence tomography angiography (OCTA) was adopted widely during the clinical practice and studies showed that OCTA is more sensitive and accurate than traditional angiography in detecting CNV in eyes with chronic CSCR [11]. Given the close association of CNV with poor visual prognosis in CSCR patients, early detection of CNV by OCTA is essential for the effective management and the follow-up of the patients. Although the resolution of OCTA images has approached the histological level, there is still a certain gap between them, which means OCTA may not be sensitive enough to detect the fibrovascular changes of incipient CNV [12]. Furthermore, our previous study found that due to the relatively low resolution of OCTA and the obscuration of SRF in some patients with CSCR, incipient or putative CNV appears as nonhomogenous hyperreflectivity in the slab of choriocapillaris on OCTA and would gradually develop into a well-defined visible CNV with peripheral anastomosis and dense microvasculature [13]. This result indicated that initial nonhomogenous hyperreflectivity might be a characteristic manifestation of incipient CNV, and these patients may benefit from early anti-VEGF therapy.

To further investigate the possible effect and efficacy of anti-VEGF therapy for CSCR patients with putative or visible CNV based on OCTA characteristics, we retrospectively reviewed 16 eyes in 15 treatment-naïve CSCR patients. Furthermore, we specifically explored the anatomical characteristics on OCTA of eyes of CSCR patients with putative CNV in an attempt to assist in finding the optimal therapeutic time window with the guidance of OCTA.

## 2. Methods

### 2.1. Subjects

This study was a retrospective cohort study (ClinicalTrials.gov number, https://www.chictr.org.cn, ChiCTR2000038911), complying with the tenets of the Declaration of Helsinki. In this retrospective cohort study, we enrolled 16 consecutive treatment-naïve eyes from 15 patients diagnosed with CSCR based on a comprehensive ophthalmologic history and examinations in the Department of Ophthalmology, Shanghai General Hospital affiliated to Shanghai Jiao Tong University, Shanghai, China, between April 2020 and February 2022. All individual participants were provided with written informed consent. The study was approved by the Clinical Research Ethical Committee of Shanghai General Hospital affiliated to Shanghai Jiao Tong University, Shanghai, China (Permit No. 2020KY205-2).

The participants underwent full ophthalmologic examinations, including visual acuity, intraocular pressure (IOP) measurement, anterior segment evaluation using slit-lamp biomicroscopy, fundus photography, and OCTA. The patients with CSCR were demonstrated with obvious SRF in b-scan of OCTA. The patients were divided into two groups based on their characteristics in the slab of choriocapillaris on OCTA, that is, (1) putative CNV group, presenting with nonhomogenous hyperreflectivity on OCTA, and (2) visible CNV group, presenting with obvious tangled vascular network on OCTA. Exclusion criteria were (1) optic media impeding sufficient image quality; (2) patients underwent thermal laser photocoagulation or verteporfin photodynamic therapy; (3) patients complicated with other eye diseases, such as age-related macular degeneration, diabetic retinopathy, glaucoma, and uveitis; and (4) any previous intraocular surgery history. The patients underwent monthly intravitreal anti-VEGF injections in the study period.

### 2.2. Intravitreal Injection of anti-VEGF Agents

The intravitreal injection was performed at the temporal limbus through the eyeball's pars plana under aseptic conditions. All patients received monthly intravitreal injections of aflibercept (2 mg/0.05 mL, 9 eyes), conbercept (0.5 mg/0.05 mL, 2 eyes), or ranibizumab (0.5 mg/0.05 mL, 5 eyes) using a 30-gauge needle. One week after intravitreal injection, the efficacy of anti-VEGF was assessed. The number of intravitreal injections was determined based on efficacy and patient's preference.

### 2.3. Optical Coherence Tomography Angiography (OCTA) Evaluation

The diagnosis of visible CNV or putative CNV secondary to CSCR was based on the image using the RTVue XR Avanti OCT system (Optovue, Inc., Fremont, CA, USA), and measurements were acquired using the manufacturer's AngioVue software (version number 2018.1.0.43). This version was an updated version of the software that used the three-dimensional (3D) projection artifact removal (PAR) algorithm. 3D PAR-enabled software was used to eliminate projection artifacts from en-face OCTA images as well as b-scan OCTA images [14,15]. The scan covered an area of 3 × 3 mm^2^ volume scan (304 × 304 b-scans) or 6 × 6 mm^2^ volume scan (400 × 400 b-scans) section centered on the fovea. The delineation was segmented on the choriocapillaris layer with OCTA, putative CNV was demonstrated as the enhanced non-homogeneous hyperreflective abnormalities in the choriocapillaris slab, which lies between the depths of 30 and 60 *μ*m below the RPE-Bruch's membrane complex, and visible CNV was demonstrated as tangled vascular network in the slab. Using the ImageJ (National Institutes of Health, Bethesda, MD) software, the CNV area was manually outlined and quantified using the polygon selection tool in the selected slab. Pigment epithelial detachment (PED) was defined as an elevation of the RPE allowing the distinct visualization of Bruch's membrane on OCTA b-scan, including the typical dome-shaped serous PEDs and flat irregular PEDs [16].

Other parameters included the changes of CMT, SRF, and HRF before and after anti-VEGF treatment. CMT was defined as the average retinal thickness between the inner limiting membrane and the inner border of RPE within the 1-mm-diameter central field of the Early Treatment Diabetic Retinopathy Study (ETDRS), which was measured automatically using the built-in software of the OCT devices. The resolution of SRF was represented by the reduction of CMT, and the height of SRF, between the outer border of the detached neurosensory retina and the inner border of the RPE, was analyzed with ImageJ. HRF detected with OCTA were defined as discrete and well-circumscribed dot-shaped lesions of equal or higher reflectivity than the RPE band, without back shadowing. HRF, with diameters ranging between 20 and 50 *μ*m [17], were considered activated microglia/macrophages in the retina [18,19]. HRF were manually counted within the length of 1,000 *μ*m centered on the fovea on b-scan of OCTA by two independent raters. There were two retinal specialists who were trained in the evaluation of OCTA images and performed all assessments.

### 2.4. Statistical Analyses

The data were analyzed by using SPSS 22.0 software. All values were presented as the number or mean ± standard deviation (mean ± SD). The visual acuity was presented as the logarithm of the minimum angle of resolution (logMAR). A *P*-value < 0.05 was considered statistical significance.

## 3. Results

### 3.1. Baseline Characteristics of Patients with CSCR

This study was a retrospective cohort study, and 16 eyes of 15 patients, including 6 females (37.5%) and 9 males (62.5%), were enrolled. The mean age was 51.1 ± 8.0 years (range 35–65 years). The average follow-up time was 3.90 ± 3.37 months. All patients were treated with anti-VEGF treatment after the first presentation, in which ten eyes (62.5%) received more than one injection. The mean number of injections was 2.69 ± 1.72, and the mean intervals between injections were 1.34 ± 0.87 months (Table 1). Among the treated eyes, 16 (100%) revealed the presence of SRF and 16 (100%) with PEDs. PEDs appeared in both the visible CNV and the presumed CNV group with no significant difference.

Among all eyes diagnosed with CSCR, visible CNV was diagnosed in 6 eyes (37.5%), while 10 eyes demonstrating nonhomogenous hyperreflectivity in the choriocapillaris layer on OCTA were diagnosed with putative CNV. There was no statistically significant difference in baseline characteristics between the two groups (Table 1).

### 3.2. Visual Acuity Improved after anti-VEGF Treatment

**Figure 1:**
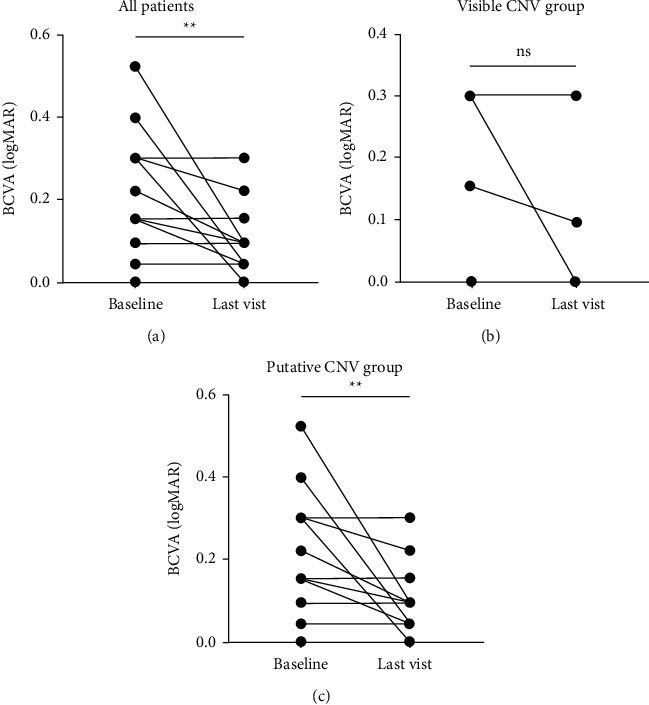
The changes of BCVA in CSCR patients at baseline and last follow-up after anti-VEGF treatment. (a) The changes of BCVA logMAR scale for all patients. (b) The changes of BCVA in logMAR scale in the visible CNV group. (c) The changes of BCVA in logMAR scale in putative CNV group. The data were represented as mean±SD. CNV, choroidal neovascularization; CSCR, central serous chorioretinopathy; logMAR, the logarithm of minimal angle of resolution. The statistics was analyzed by paired t-test; ns, not statistically significant; ^*∗*^*P* < 0.05 and ^*∗∗*^*P* < 0.01.

After intravitreal injection of anti-VEGF reagents, the visual acuity (logMAR) improved significantly from 0.189 ± 0.145 (baseline) to 0.087 ± 0.081 (last visit, *n* = 16, *P* = 0097) (Figure 1(a)). Interestingly, visual acuity showed no significant difference in the visible CNV group between the baseline and the last follow-up (*n* = 6, *P* = 0.2052) (Figure 1(b)), but improved significantly in the putative CNV group (*n* = 10, *P* = 0.0319) (Figure 1(c)).

### 3.3. Central Macular Thickness (CMT) Was Decreased and Subretinal Fluid (SRF) Was Resolved after anti-VEGF Treatment

**Figure 2:**
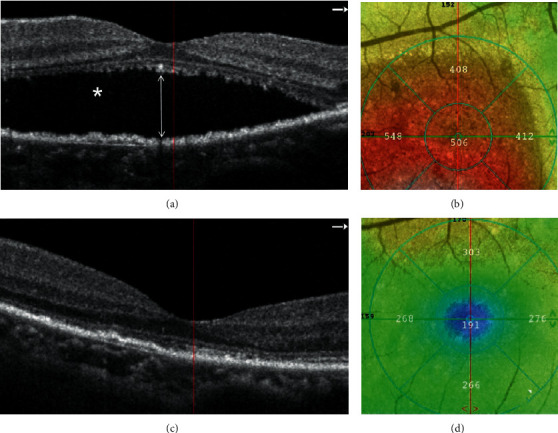
Representative image of the patient with CSCR showing CMT reduction and SRF resolution after anti-VEGF treatment. An 49-year-old female with CSCR. Her initial BCVA (logMAR) was 0.30, which was improved to 0.05 after 3 consecutive intravitreal injections of aflibercept. (a) OCTA image (3∗3 mm^2^) showed the presence of SRF (asterisk) and the height of SRF (double-head arrow); (b) The CMT before treatment was about 506 *μ*m; (c) Complete resolution of SRF after three anti-VEGF treatments. (d) The CMT was about 191 *μ*m after the final follow-up.

**Figure 3:**
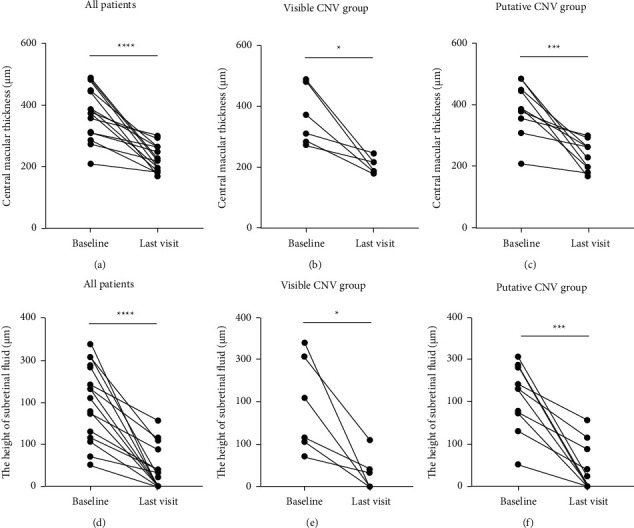
The quantitative analysis of CMT and SRF in CSCR patients with visible or putative CNV before and after anti-VEGF treatment. (a) The changes of CMT for all the patients. (b) The changes of CMT in the visible CNV group. (c) The changes of CMT in the putative CNV group. (d) The changes of the height of SRF for all the patients. (e) The changes of the height of SRF in the visible CNV group. (f) The changes of the height of SRF in the putative CNV group. The data were represented as mean ± SD; CMT, central macular thickness; SRF, subretinal fluid. The statistics was analyzed by the paired *t*-test, ^*∗∗*^*P* < 0.01, ^*∗∗∗*^*P* < 0.001, and ^*∗∗∗*^*P* < 0.0001.

Both CMT and SRF were reduced significantly after anti-VEGF injections (Figures 2 and 3). At the last follow-up, CMT was reduced from 381.77 ± 84.10 *μ*m to 225.09 ± 41.17 *μ*m (*n* = 16, *P* < 0.0001) and the height of SRF was reduced from 204.77 ± 86.90 *μ*m to 38.25 ± 50.01 *μ*m (*n* = 16, *P* < 0.0001) (Figures 3(a) and 3(d)). Complete resolution of SRF was found in 8 out of 16 eyes during the follow-up, in which 5 eyes were putative CNV (Figures 3(e) and 3(f)).

### 3.4. The Area of Putative CNV Was Decreased after Anti-VEGF Treatment

**Figure 4:**
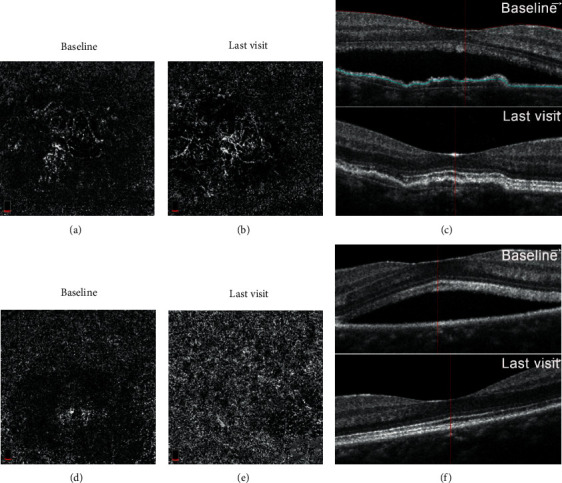
The outcome of both eyes in the same patient after anti-VEGF treatment. (a–c) The right eye. Visible CNV with peripheral anastomosis and dense microvasculature visualized in the choriocapillaris layer on OCTA before (a) and after (b) anti-VEGF treatment, showing vascular remodeling after anti-VEGF treatment. (c) OCTA b-scan showed the resolution of SRF and irregular pigment epithelial detachment (PED) before and after anti-VEGF treatment. (d–f) The left eye. Nonhomogenous hyperreflectivity (putative CNV) in the choriocapillaris layer on OCTA before (d) and after (e) anti-VEGF treatment, showing complete regression of putative CNV after treatment; (f) OCTA b-scan showed complete resolution of SRF after anti-VEGF treatment.

Figure 4 shows the difference between both eyes detected with OCTA in a 56-year-old female, who was diagnosed with CSCR. In her right eye, there was a well-defined CNV (visible CNV) with peripheral anastomosis and dense microvasculature in the slab of choriocapillaris on OCTA (Figure 4(a)), while her left eye revealed the nonhomogenous hyperreflectivity (putative CNV) in the choriocapillaris layer on en-face of OCTA (Figure 4(d)). With anti-VEGF treatment, vascular remodeling was seen in her right eye (Figure 4(b)), while nonhomogenous hyperreflectivity was diminished obviously (Figure 4(e)). SRF in both eyes was resolved completely after anti-VEGF treatment (Figures 4(c) and 4(f)), suggesting anti-VEGF treatment was effective for CSCR patients with visible or putative CNV.

**Figure 5:**
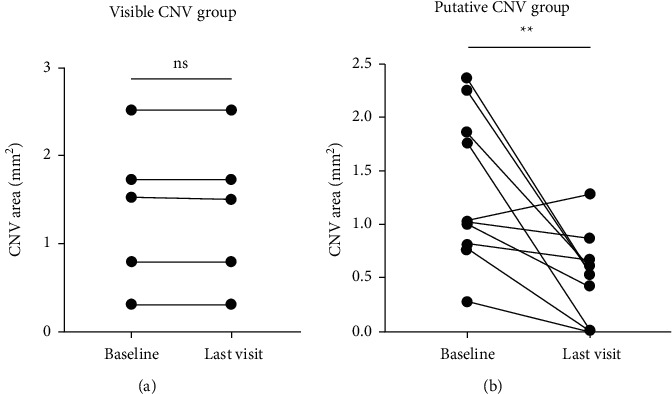
The changes of visible or putative CNV area at baseline and at the final follow-up after anti-VEGF treatment. (a) The changes of CNV area in the visible CNV group. (b) The changes of putative CNV area in the putative CNV group. The data were represented as mean±SD; CNV, choroidal neovascularization. The statistics was analyzed by paired t-test, ns, not statistically significant; ^*∗∗*^*P* < 0.01.

**Figure 6:**
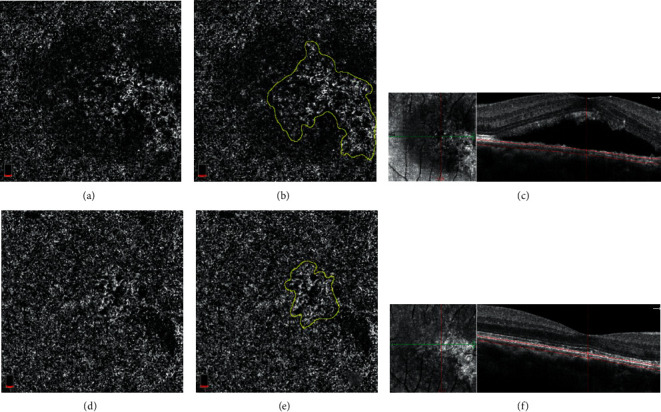
Representative image showing the decrease of putative CNV area and resolution of SRF in CSCR patient before and after anti-VEGF treatment. A 50-year-old female patient, who was diagnosed as CSCR with putative CNV in right eye, underwent three consecutive intravitreal injection of anti-VEGF treatments. (a–c) Baseline (before treatment). (a) The putative CNV was visualized as blurred nonhomogeneous hyperreflective signals on the slab of the choriocapillaris on OCTA; (b) The putative CNV in Figure a was outlined using ImageJ to calculate its area; (c) The corresponding OCTA b-scan showed the presence of SRF. (d–f) Treatment (after anti-VEGF treatment). (d) The regression of putative CNV after 3 consecutive anti-VEGF (aflibercept) treatments; (e) The regressed putative CNV area in Figure d was outlined using ImageJ; (f) OCTA b-scan showed complete resolution of SRF after three anti-VEGF treatments.

Furthermore, we compared the area changes in both the CNV group and the putative CNV group. Compared with the baseline, the CNV area remained relatively unchanged between the baseline and the last visit in the visible CNV group (*n* = 6, *P* = 0.4015) (Figure 5(a), while in the putative CNV group, the putative CNV area was markedly declined from 1.32 ± 0.66 mm^2^ (baseline) to 0.50 ± 0.40 mm^2^ (last visit, *n* = 10, *P* = 0.0081) (Figure 5(b))). To be consistent with the results of Figure 5(b), the putative CNV area together with the reduction of SRF was declined remarkedly in one CSCR patient, who was demonstrated with nonhomogenous hyperreflectivity in the choriocapillaris layer in OCTA at baseline and underwent 3 consecutive intravitreal injections of aflibercept, indicating that the early initiation of anti-VEGF treatment is of importance for CSCR patients coexisting putative CNV (Figure 6).

### 3.5. Hyperreflective Foci (HRF) in the Outer Retina and Choriocapillaris Were Decreased after Anti-VEGF Treatment

**Figure 7:**
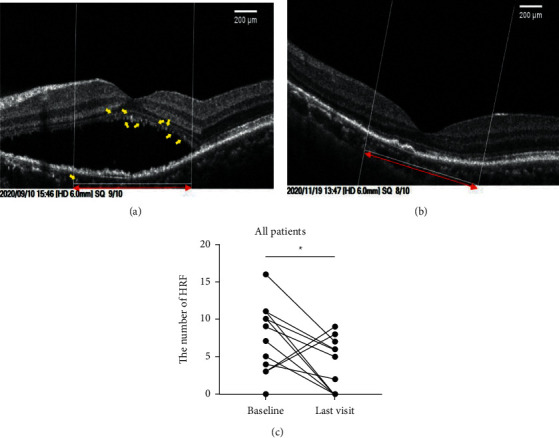
Representative image showing the change of HRF in patients with CSCR before and after anti-VEGF treatment. A 50-year-old female with CSCR, who was diagnosed with CSCR with visible CNV in the left eye, underwent three consecutive intravitreal injection of anti-VEGF treatments. (a) The baseline OCTA image showing SRF and HRF in his right eye. HRF (yellow arrow) were measured within the length of 1,000 *μ*m (red double-head arrow) between 2 vertical lines centered on the fovea. (b) The number of HRF was decreased accompanying partial SRF resolution after anti-VEGF treatment. (c) The changes of HRF for all the patients. HRF, hyperreflective foci; SRF, subretinal fluid. The statistics was analyzed by the paired *t*-test, ^*∗*^*P* < 0.05.

The changes of HRF were measured before and after anti-VEGF treatment, demonstrating the decreasing number of HRF after treatment (Figure 7). Compared with baseline (Figure 7(a)), anti-VEGF effectively reduced the height of SRF and the number of HRF (Figure 7(b)). The mean number of HRF was decreased from 5.56 ± 4.95 to 2.69 ± 3.35 in the outer retina and choriocapillaris layer (*n* = 16, *P* = 0.0343) (Figure 7(c)). The number of HRF was declined in almost all patients except two patients who underwent only one intravitreal injection.

### 3.6. Adverse Effects

No side effects were observed after anti-VEGF treatments.

## 4. Discussion

In our previous observation, we found that the nonhomogenous hyperreflectivity in the slab of choriocapillaris on OCTA would generally develop into visible CNV in patients with CSCR during the follow-up visit [13]. Thus, we proposed that the nonhomogenous hyperreflectivity might be a sign of the early state of the proliferation of vascular endothelial cells, which represented putative or incipient CNV, and demanded the early anti-VEGF treatment for possible favorable outcomes. In the present study, we defined CSCR patients with nonhomogenous hyperreflectivity in the slab of choriocapillaris on OCTA as CSCR coexisting putative CNV and compared the potential effect and efficacy of anti-VEGF treatment in both the visible CNV group and the putative CNV group, thus to propose the optimal timing of treatment for CSCR patients based on OCTA evaluation.

Risk factors for CSCR with coexisting CNV include chronic CSCR, female gender, choroidal hyperpermeability, poor visual acuity at the first visit, hypertension, and flat irregular PED (double layer signs). [6, 20]. Although CNV is one of the most severe complications of CSCR, it is challenging to diagnose CNV in CSCR because CSCR itself can be associated with SRF, PED, and ill-defined patterns of hyperfluorescence on fluorescein angiography (FA), which largely reduce the accuracy of CNV diagnosis. Previous studies demonstrated that OCTA may be more sensitive and superior to FA or other dye-based angiography in the diagnosis of CNV in CSCR [21]. With the aid of OCTA, putative CNV secondary CSCR could be imaged and outlined with the characteristic SRF in the fovea and disturbed as the signal of hyperreflective areas in the choriocapillaris layer beneath SRF [22]. The putative CNV was supported in these patients because after anti-VEGF therapy, the putative CNV area and SRF were markedly reduced (Figure 3(f); Figure 5(b)). Nearly all patients in the putative CNV group (7/10) had complete or near-complete resolution of SRF after injections. Although the visible CNV area did not decrease significantly, the putative CNV area, that is, nonhomogenous hyperreflectivity in the slab of choriocapillaris on OCTA, was decreased significantly by anti-VEGF treatment (Figure 5). The resolution of SRF might indicate the inhibitory effect or regression of neovascularization by anti-VEGF drugs on the mature CNV or incipient CNV to reduce the active leakage of CNV through the hyperpermeable RPE into the subretinal space. The findings also suggested that a lag existed between restoration of choroidal vascular permeability and regression of CNV by anti-VEGF mechanism. In fact, when eyes with relatively mature neovessels (visible CNV) were excluded, an obvious reduction of putative CNV area was observed from 1.32 ± 0.66 mm^2^ (baseline) to 0.50 ± 0.40 mm^2^ (last visit), indicating that relatively mature neovessels were less responsive to anti-VEGF therapy, which was comparable to the published study [23]. The may imply the initiation of early anti-VEGF treatment for CSCR patients with putative or visible CNV.

We further explored how anti-VEGF therapy might play a role in the treatment of CSCR patients with putative or visible CNV. Research studies showed the following mechanisms were involved in the formation of SRF in CSCR coexisting putative or visible CNV, including the breakdown of the outer blood-retinal barrier (BRB), the drainage dysfunction of RPE, the involvement of inflammatory cells (microglia, macrophages), and the release of pro-inflammatory mediators, including VEGF, placental growth factor (PGF), interleukin-1 beta (IL-1*β*), intercellular cell adhesion molecule-1 (ICAM-1), and monocyte chemoattractant protein-1 (MCP-1). [24, 25]. A recent study confirmed that patients with CSCR have imbalanced plasma levels of VEGF and pigment-epithelium-derived factor (PEDF) [26]. VEGF and PGF are potent pro-angiogenic factors, causing the breakdown of BRB, drainage dysfunction of RPE, and promoting the formation of CNV [27]. There is no clear evidence that anti-VEGF could improve the cellular drainage function of RPE. Recently, we showed that anti-VEGF treatment could improve the drainage function of the Müller cell in experimental diabetic retinopathy [28]. Anti-VEGF drug (ranibizumab) protected Müller cells from intracellular edema through the upregulation of Kir4.1 and AQP4 by directly binding VEGF-A, increasing Na^+^-K^+^-ATPase expression, and decreasing the intracellular osmotic pressure of Müller cells [28]. The possible mechanisms to decrease intracellular edema in Müller cells might be extrapolated to RPE exerted by anti-VEGF reagents.

Besides, both VEGF and PGF could also induce inflammation by activating microglia/macrophages and promoting the release of inflammatory factors [29]. It was proposed that HRF in the outer retina might correspond to the activated microglia and/or macrophages, indicating that inflammation was involved in the pathogenesis and development of CSCR [30]. Besides CSCR, HRF were also reported in other eye diseases, such as diabetic macular edema, neovascular age-related macular degeneration, and retinitis pigmentosa. [28, 31, 32]. In the present study, a significant decrease in the HRF number was also observed in CSCR patients after anti-VEGF treatment (Figure 7). Since HRF on OCT or OCTA b-scans are considered activated microglia/macrophages, the decreased number of HRF found in the current study indicated that anti-VEGF agents also have the anti-inflammatory function to deactivate microglia and/or macrophages, which merits further exploration of the detailed mechanisms. Moreover, in our study, HRF were mainly distributed in the outer retina and the choriocapillaris layer, which was decreased after anti-VEGF treatment, accompanied with the improvement of visual acuity and resolution of SRF. Thus, the reduction in the HRF number detected by OCTA heralds a favorable anatomical and functional outcome after anti-VEGF treatment.

**Figure 8:**
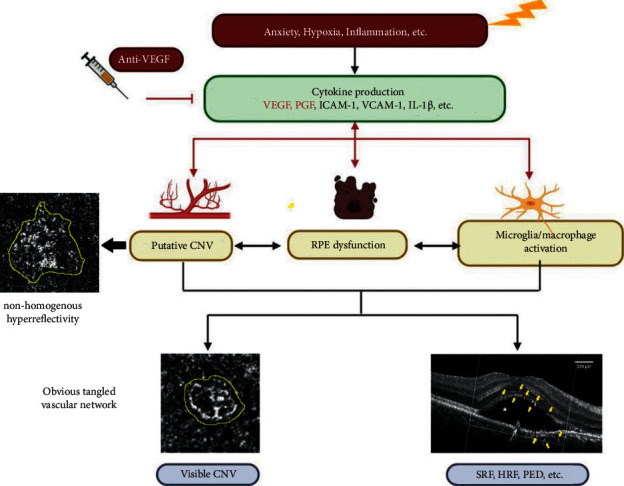
Schematic diagram depicting the progression and potential mechanism of CSCR coexisting putative or visible CNV and the possible effect of anti-VEGF treatment. CNV, choroidal neovascularization; CSCR, central serous chorioretinopathy; HRF, hyperreflective foci (yellow arrow); ICAM-1, intercellular cell adhesion molecule-1; PGF, placental growth factor; RPE, retinal pigment epithelium; SRF, subretinal fluid (asterisk); PED, pigment epithelial detachment (white arrow); VCAM-1, vascular cell adhesion molecule-1; VEGF, vascular endothelial growth factor. The putative CNV and visible CNV were circled with dotted yellow circle on the choriocapillaris layer in OCTA.

Overall, we inferred that putative or incipient CNV in patients with CSCR existed in the form of nonhomogenous hyperreflectivity in the choriocapillaris layer. The relatively low resolution and insufficient magnification of the OCTA images did not allow for the detection of fine microvascular structures when compared to histopathology for CNV detection, which may partially explain why putative CNVs in OCTA appeared as nonhomogenous hyperreflectivity rather than ring-like or strip-like structures [13]. In Figure 8, we proposed the possible pathogenesis and progression of CSCR based on the current observations. Under certain conditions, such as hypoxia in outer retina or RPE-Bruch's membrane-choriocapillaris complex (RBCC), the cytokines including VEGF, PGF, ICAM-1, and VCAM-1 were upregulated. These upregulated cytokines, for example, VEGF and PGF, can affect different cells in both retina and choroid as follows: (1) induce RPE dysfunction leading to increased permeability of the outer BRB and decreased drainage function of RPE, resulting in the formation of SRF in OCTA b-scan; (2) induce the proliferation, migration, and tube formation of choriocapillaris endothelial cells, which increased the leakage of neovessels in choriocapillaris through the hyperpermeable RPE into subretinal space, aggregating SRF; and (3) activate the inflammatory cells, such as microglia and macrophage, inducing their proliferation, migration, and secretion of more inflammatory factors or growth factors, which participate and complicate the pathogenesis of CSCR (Figure 8). The proliferation and tube formation of choroidal vascular endothelial cells are actually immature neovessels, which are displayed as nonhomogenous hyperreflectivity in the choriocapillaris layer in OCTA and designated as putative CNV in our study; with disease progression, the putative CNV evolves into visible CNV demonstrating obvious tangled vascular network in OCTA; thus, early treatment with anti-VEGF reagents or other potential treatments is of great importance to reduce the neovascularization in CSCR coexisting putative CNV and prevent its further progression.

The limitations of this study include a small sample size, the lack of a control group, and short-term observation and follow-up. Therefore, large sample multicenter studies are needed to clarify the long-term efficacy of anti-VEGF reagents in the treatment of CSCR with visible or putative CNV. The effect of different anti-VEGF drugs, such as bevacizumab, ranibizumab, afilbercept, and conbercept, should be compared with a long-term sequential follow-up. Other limitation is that self-limiting effects for the resolution of SRF cannot be ruled out even in chronic CSCR. Besides, OCTA cannot detect an active leakage as observed by FA. Thus, further studies combining OCTA with other angiographic modalities, for example, FA and indocyanine green angiography (ICGA), are required.

## 5. Conclusion

Anti-VEGF therapy appears to be a promising alternative strategy to treat CSCR with putative or visible CNV. Early initiation of anti-VEGF treatment would benefit the patients more with favorable anatomical and functional outcomes, such as visual acuity improvement, CMT reduction, SRF resolution, and decreased HRF. A long-term randomized controlled trial for CSCR patients with putative or visible CNV on OCTA treated with anti-VEGF drugs warrants further study.

## Figures and Tables

**Table 1:** Baseline characteristics of all eyes included in the study.

Criteria	All eyes	Visible CNV group	Putative CNV group	*P*-value
Patients (n)	16	6	10	—
Age (mean ± SD)	51.1 ± 8.0	51.9 ± 8.1	50.6 ± 7.9	0.762
Gender (male/Female)	9/6	3/3^*∗*^	6/4^*∗*^	0.696
Mean interval of follow-up (months) (mean ± SD)	3.90 ± 3.37	4.29 ± 3.42	3.67 ± 3.32	0.743
Affected eye (right/Left)	7/9	3/3	4/6	0.696
The number of injections (mean ± SD)	2.69 ± 1.72	2.67 ± 2.13	2.70 ± 1.42	0.973
Mean interval between injections in months (mean ± SD)	1.34 ± 0.87	1.42 ± 0.99	1.29 ± 0.78	0.716
Type of fluid accumulation (Subretinal/PED)	16/16	6/6	10/10	—

CNV, choroidal neovascularization; PED, pigment epithelial detachment. The data were represented as mean ± SD; ^*∗*^ one patient with both eyes affected. The statistics was analyzed by the chi-square test or independent *t*-test when appropriated.

## Data Availability

The data that support the findings of this study are available from the corresponding author upon reasonable request.
